# Impact of premature mortality attributable to obesity on years of life lost in Brazil

**DOI:** 10.20945/2359-4292-2026-0087

**Published:** 2026-08-05

**Authors:** Luís Jesuino de Oliveira Andrade, Gabriela Correia Matos de Oliveira, Osmario Jorge de Mattos Salles, Alcina Maria Vinhaes Bittencourt, Luís Matos de Oliveira

**Affiliations:** 1 Departamento de Saúde, Universidade Estadual de Santa Cruz, Ilhéus, BA, Brasil; 2 Hospital Electro Bonini e Maternidade Cidinha Bonini, Universidade de Ribeirão Preto, Ribeirão Preto, SP, Brasil; 3 Escola Bahiana de Medicina e Saúde Pública, Salvador, BA, Brasil; 4 Faculdade de Medicina, Universidade Federal da Bahia, Salvador, BA, Brasil

**Keywords:** Obesity, years of life lost, premature mortality

## Abstract

**Objective:**

To quantify the societal burden of obesity in Brazil by estimating years of life lost (YLL) due to premature mortality.

**Subjects and methods:**

Given the Brazilian life expectancy of 76.4 years, premature death was defined as occurring before the age of 60. A quantitative epidemiological approach was applied to estimate obesity-attributable YLL in Brazil from 2014 to 2023. Mortality data were obtained from the Mortality Information System, and demographic projections were sourced from the Brazilian Institute of Geography and Statistics. The YLL was calculated by multiplying the number of deaths by the remaining life expectancy (using 2021 Global Burden of Disease reference tables), stratified by age and sex. Statistical analyses were conducted using R software and Python, with results presented as rates per 100,000 population.

**Results:**

The total number of events exhibited an upward trend over the decade, with a pronounced peak after 2020, coinciding with the COVID-19 pandemic. The 50-59 and 60-69-year age groups consistently demonstrated the highest event frequencies. Sex-specific analyses revealed variations across age groups and years. Events were rare among those aged 1-14 but increased from age 15-19 onwards. Among older age groups (40-69 years), females exhibited higher rates than males, particularly in the 60-69-age bracket.

**Conclusion:**

This study demonstrates an increasing trend in obesity-related premature mortality in Brazil, disproportionately affecting older populations and necessitating targeted public health interventions.

## INTRODUCTION

Obesity has emerged as one of the most pressing global public health challenges, with its prevalence rising dramatically in both high-income and lowand middle-income countries (^1^). Defined by excessive body fat accumulation, obesity is a major contributor to premature mortality through its association with cardiovascular diseases, type 2 diabetes mellitus, at least 13 types of cancer, respiratory diseases, musculoskeletal disorders, chronic kidney disease, liver disease, and numerous other conditions (^2^). In Brazil, obesity prevalence has increased over the past 50 years, affecting individuals across all age groups and socioeconomic strata (^3^). This escalating issue is compounded by regional disparities in healthcare access, socioeconomic inequalities, and the double burden of malnutrition (^4^). Quantifying the societal impact of obesity-related deaths requires robust epidemiological metrics, such as years of life lost (YLL), which estimate the gap between observed age at death and standard life expectancy (^5^).

YLL serves as a critical metric to contextualize the burden of premature mortality, emphasizing the societal cost that extends beyond traditional mortality statistics (^6^). Studies utilizing YLL calculations have revealed up to 40% higher disease impacts than conventional mortality analyses, underscoring the necessity for region-specific assessments (^7^). Socioeconomic determinants play a pivotal role in obesity outcomes in Brazil, with income inequality and limited healthcare access contributing to differential mortality rates (^8^). Individuals from lower socioeconomic backgrounds face higher risks of obesity-related complications and premature death, attributable to reduced access to healthy foods and limited opportunities for physical activity (^9^).

Temporal trends reveal concerning patterns, as national surveillance data indicate a 45% increase in obesity-related mortality between 2010 and 2020 (^10^). The COVID-19 pandemic significantly disrupted healthcare services, including limited access to bariatric surgery and clinical follow-up in both public and private healthcare systems, and may have exacerbated premature mortality among individuals with obesity, though findings are not entirely consistent across studies (^11^-^13^). Given this context, this study sought to quantify the societal burden of obesity in Brazil by estimating YLL due to premature mortality between 2014 and 2023, providing age-, sex-, and time-stratified estimates to inform evidence-based policy development aligned with Sustainable Development Goal target 3.4 (^14^).

## SUBJECTS AND METHODS

This study utilized a quantitative epidemiological framework to estimate the burden of premature mortality attributable to obesity in Brazil. The core measure was YLL, which estimates the average years a person would have lived if not for premature death due to obesity-related causes.

### Data collection

Mortality data were retrieved from the Mortality Information System maintained by the Brazilian Ministry of Health, covering 2014 to 2023. The dataset was disaggregated by age, sex, and cause of death. Selection criteria included records in which obesity was documented as either the underlying or a contributing cause of death, using International Classification of Diseases, Tenth Revision (ICD-10) codes: E66.0 (obesity due to excess calories), E66.1 (drug-induced obesity), E66.2 (extreme obesity with alveolar hypoventilation), E66.8 (other forms of obesity), and E66.9 (obesity, unspecified). Importantly, obesity-related mortality is likely underestimated on death certificates, as obesity itself is frequently omitted, while obesity-associated conditions (e.g., cardiovascular or metabolic diseases) are commonly listed as causes. The principal underlying causes associated with obesity included diseases of the circulatory system (I00-I99), particularly ischemic heart diseases (I20-I25) and cerebrovascular diseases (I60-I69); endocrine and metabolic disorders, especially type 2 diabetes mellitus (E11); malignant neoplasms (C00-C97); and diseases of the respiratory system (J00-J99). This refined case definition enhances the etiological specificity of mortality patterns attributable to obesity in Brazil. During the COVID-19 pandemic, inconsistent documentation of anthropometric measurements further limited the precise assessment of adiposity status. Demographic information, including population size and age-sex distribution, was obtained from the Brazilian Institute of Geography and Statistics to accurately characterize the population at risk.

### Calculation of years of life lost

YLL was calculated by multiplying the number of deaths within specific age and sex strata by the corresponding remaining life expectancy at the age of death. Life expectancy references were adopted from the 2021 Global Burden of Disease (GBD) study (^15^), ensuring standardized and internationally comparable benchmarks. Age weighting and time discounting were not applied, in keeping with current ethical considerations and best practices.

### Data analysis

Data processing and statistical analyses were performed using the open-source software PSPP and the Python programming language, with additional calculations in Excel. Mortality rates were standardized per 100,000 population and were further stratified by age and sex to assess trends and disparities over the study period.

### Contextual factors

Socioeconomic and healthcare variables such as income distribution, access to health services, and public health policies relevant to obesity prevention and management were incorporated from governmental databases and published literature, providing a comprehensive understanding of the social determinants influencing premature mortality related to obesity.

### Ethical considerations

Given the exclusive use of aggregated, anonymized, publicly available data, this study was exempt from individual consent or institutional ethical review, in compliance with Brazilian regulations governing secondary data research.

## RESULTS

### Obesity-related premature mortality in Brazil (2014-2023)

Premature death was defined as occurring before age 60, in the context of a national life expectancy of 76.4 years. The YLL was calculated by multiplying deaths in each age-sex stratum by the remaining life expectancy from the GBD study (^15^). Results are reported as age-standardized rates per 100,000 population.

### Temporal trends in obesity-attributable mortality

Obesity-related deaths were observed throughout the study period, with a marked acceleration following 2020, temporally coinciding with the onset of the COVID-19 pandemic. Annual deaths increased from 1,900 in 2014 to a peak of 3,488 in 2021, then declined modestly to 2,660 in 2023 (**Table 1)**. This pattern suggests a pandemic-related disruption in chronic disease management and healthcare access that may have exacerbated underlying obesity-related pathologies. The analysis indicated that, while the pandemic amplified mortality, the long-term trend primarily reflects ongoing epidemiological transitions rather than an isolated infectious event.

**Table 1:** Annual distribution of obesity-attributable deaths by age group, Brazil

Year	Age range									Total
	01-04	05-09	10-14	15-19	20-29	30-39	40-49	50-59	60-69	
2014	1	2	3	11	102	287	439	548	507	1,900
2015	2	1	4	16	89	264	361	579	610	1,926
2016	0	2	4	12	102	309	489	625	541	2,084
2017	2	1	2	10	97	270	469	600	603	2,054
2018	2	3	2	10	101	307	531	616	658	2,230
2019	1	0	0	10	108	341	550	679	691	2,380
2020	1	0	1	17	136	447	671	779	838	2,890
2021	1	1	4	18	160	488	862	964	990	3,488
2022	2	5	5	23	132	369	617	744	781	2,678
2023	3	2	5	19	138	367	674	742	710	2,660

Source: TABNET - Brazilian Ministry of Health.

### Age distribution of mortality events

**Figure 1:**
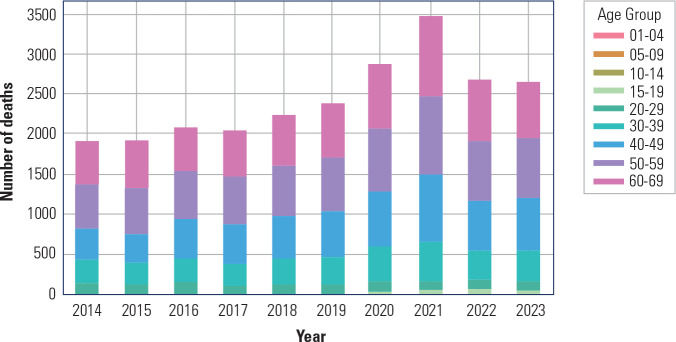
Trends in obesity-related premature mortality by age group.

Mortality events were negligible among children and adolescents (ages 1-14), with fewer than five deaths annually in this group. Event frequency increased progressively from age 15 onward, with the highest burden concentrated in the 50-59 and 60-69 age groups. These two strata consistently accounted for over 50% of all obesity-attributable deaths each year, underscoring the disproportionate impact on middle-aged and older adults (**Figure 1**).

### Sex-specific disparities

**Figure 2:**
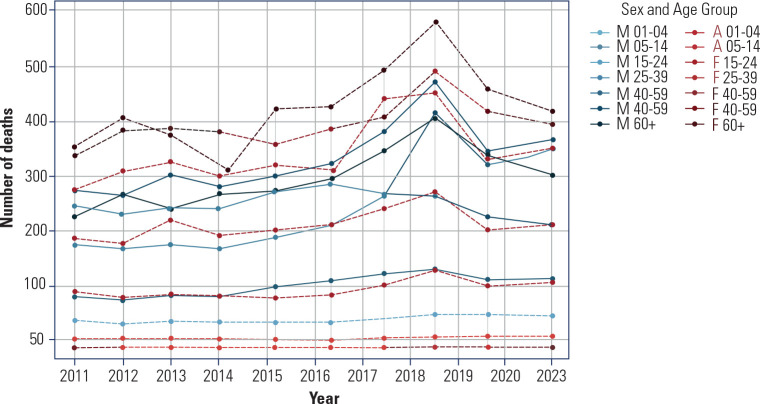
Sex-specific trends in obesity-attributable YLL across age groups

Sex-stratified analysis revealed notable differences in mortality patterns. Males had slightly higher death counts in younger adult groups (20-49 years), while females consistently surpassed males in the 50-69 age brackets **(Figure 2).** This disparity was most pronounced in the 60-69 group, where female deaths exceeded male deaths every year, reaching a difference of 188 additional female deaths in 2021 (**Table 2**). These findings suggest a sex-based divergence in obesity-related disease progression, healthcare-seeking behavior, or biological susceptibility during later life stages.

**Table 2:** Sex-disaggregated counts of obesity-attributable deaths by age group and year

Age range	Male-female								
	01-04	05-09	10-14	15-19	20-29	30-39	40-49	50-59	60-69
2014	1-0	2-0	3-0	8-3	47-55	138-149	206-233	244-304	186-321
2015	0-2	1-0	2-2	9-7	43-46	127-137	188-273	222-357	231-379
2016	0-0	1-1	2-2	8-4	50-52	134-175	200-289	267-358	197-344
2017	1-1	1-0	2-0	4-6	49-48	125-145	200-269	245-355	231-272
2018	1-1	2-1	1-1	5-5	53-48	146-161	239-292	278-338	240-418
2019	1-0	0-0	0-0	5-5	64-44	170-171	259-291	304-375	268-423
2020	1-0	0-0	0-1	10-7	77-59	234-213	235-436	374-405	340-498
2021	1-0	1-0	2-2	7-11	80-80	241-247	411-451	474-490	401-589
2022	1-1	3-2	5-0	10-13	71-61	194-175	301-316	333-411	325-456
2023	1-2	1-1	2-3	8-11	73-65	184-183	336-338	353-389	287-423

Source: TABNET - Brazilian Ministry of Health.

Overall, this study demonstrated a sustained upward trend in obesity-attributable premature mortality across Brazil from 2014 to 2023, with the highest burden among middle-aged and older adults. A pronounced gender disparity was observed, as women exhibited consistently higher mortality rates than men in advanced age groups, suggesting sex-specific vulnerabilities in obesity-related health outcomes.

## DISCUSSION

Our study demonstrated an escalation in obesity-driven premature mortality throughout Brazil, disproportionately affecting older demographics and revealing substantial sex-based vulnerabilities that necessitate urgent, evidence-based public health interventions.

Analysis of Mortality Information System data revealed that the main causes of death associated with obesity during the study period were predominantly cardiovascular diseases (including ischemic heart disease, stroke, and heart failure), type 2 diabetes mellitus and its complications, certain cancers (particularly those of the digestive system, breast, and reproductive organs), chronic respiratory diseases, and chronic kidney disease (^16^). These findings are consistent with established international evidence on obesity-related mortality, reinforcing that most premature deaths attributed to obesity occur through these well-characterized pathophysiological pathways rather than obesity itself being listed as the primary cause of death (^17^).

YLL offers a time-based metric that quantifies premature death by calculating the difference between age at death and standard life expectancy for that age, thereby avoiding arbitrary age thresholds and ensuring that all deaths contribute proportionally to burden estimates (^18^). For obesity, YLL calculations revealed significant variations across demographic strata, with younger adults experiencing greater life-year losses than older populations when facing comparable obesity severity (^15^). Methods such as those from the GBD calculate YLL by multiplying cause-specific deaths across age, sex, and location by the corresponding standard life expectancies from reference tables (^19^). These estimations have shown that severe obesity results in aggregate burdens exceeding ninety-five million years lost annually, with considerable contributions from certain demographic groups (^20^). Our study aligns with the established GBD (^15^) protocol, employing standardized life expectancy references and adapting operational thresholds to Brazilian demographic realities, thereby ensuring both international comparability and local epidemiological relevance.

The sustained upward trajectory in obesity-related mortality reflects broad epidemiological transitions: non-communicable chronic diseases (NCDs) have increasingly overshadowed infectious diseases as leading causes of death, particularly among aging populations burdened by cumulative metabolic dysfunction (^21^). Globally, obesity prevalence has tripled since 1975, with projections suggesting most adults will be overweight or obese by 2050, disproportionately impacting lowand middle-income countries (^22^). Our findings align with global trends linking rising obesity mortality to shifts favoring NCDs, while also revealing a distinct pandemic-associated acceleration, suggesting that disruptions in care during this period intensified pre-existing metabolic vulnerabilities, particularly in populations already facing systemic healthcare inequities. Thus, while international transitions favor chronic metabolic diseases over infectious causes, Brazil’s trajectory demonstrates how health system disruptions can exacerbate established vulnerabilities in resource-constrained settings.

The disproportionate increase in obesity-related mortality observed during the COVID-19 period (2020-2021) warrants discussion. Multiple studies demonstrate that individuals with obesity faced higher risks of severe COVID-19 outcomes, including hospitalization, intensive care unit admission, mechanical ventilation, and death (^11^-^13^). The mechanisms underlying this vulnerability are multifactorial, including impaired immune response, chronic low-grade inflammation, reduced respiratory capacity due to chest wall restriction, and higher prevalence of comorbidities (e.g., diabetes and cardiovascular disease) (^23^). Additionally, the COVID-19 pandemic caused substantial disruptions to routine healthcare services, with postponements of elective bariatric surgeries, reduced access to nutritional counseling and weight management, and less physical activity due to lockdown measures (^24^). These factors likely contributed to the observed acceleration in obesity-attributable mortality during 2020-2021, representing both direct effects of SARS-CoV-2 infection in individuals with obesity and indirect effects from healthcare system disruptions.

Recent evidence underscores sex-specific vulnerabilities in obesity-related premature mortality, with men consistently exhibiting higher age-standardized YLL rates than women, potentially driven by differences in fat distribution, healthcare-seeking behaviors, and hormonal influences (^25^). Women, however, experience a disproportionate psychopathological burden and obesity-related malignancies, facing elevated mortality risks despite more frequent treatment-seeking compared to men (^26^). Our findings diverge from global patterns by revealing an unexpected female predominance in advanced age groups, rather than the typical male excess mortality. This inversion suggests distinctive Brazilian sociocultural factors, healthcare access barriers, or hormonal transitions uniquely amplifying women’s vulnerability.

Analyzing trends in obesity-related mortality rates excluding the COVID-19 period (2020-2021) provides further insight. Between 2014 and 2019, deaths increased from 1,900 to 2,305, a 21.3% increase over five years, with an average annual growth rate of approximately 4.0%. Post-pandemic (2022-2023), mortality rates declined from the 2021 peak but remained higher than pre-pandemic levels (2,872 deaths in 2022 and 2,660 in 2023), suggesting only partial recovery. This indicates that while the COVID-19 pandemic exacerbated obesity-related mortality, an underlying upward trend was already established in pre-pandemic years. The persistence of elevated mortality rates in 2022-2023, compared to 2019, suggests potentially lasting negative effects on obesity outcomes, possibly due to delayed diagnoses, treatment interruptions, or worsening of metabolic health during lockdowns. This mirrors global findings where obesity-related mortality remained elevated post-pandemic, especially in high-income nations, suggesting systemic healthcare disruptions persisted beyond acute COVID-19 phases (^27^).

Limitations of this study include potential underascertainment of obesity-related deaths due to inconsistent coding practices on death certificates, where obesity is often omitted as a cause. Additionally, defining premature mortality as death before age 60, despite Brazil’s life expectancy of 76.4 years, may underestimate YLL in older adults. Other limitations include reliance on secondary mortality data, introducing potential misclassification, particularly regarding the role of obesity as underlying versus contributing cause. Confounding variables including smoking, socioeconomic stratification, and comorbidity profiles remain unaccounted within aggregated datasets, potentially overestimating the direct attribution to obesity while obscuring complex causal pathways mediating YLL outcomes.

In summary, our study provides a decade-long quantification of obesity-attributable YLL in Brazil, revealing a concerning upward mortality trend, exacerbated post-2020. Notably, identifying paradoxical female predominance in advanced age groups challenges established international patterns, warranting further research into Brazil-specific sociocultural and healthcare determinants.

In conclusion, this study quantified the burden of obesity-attributable premature mortality in Brazil, revealing sustained temporal escalation with pandemic-era acceleration. Pronounced age-stratified patterns emerged, concentrating mortality among middle-aged and older individuals, with a paradoxical female predominance in advanced age brackets that challenges conventional epidemiological paradigms, requiring targeted interventions to address sex-specific vulnerabilities and reduce preventable years of life lost.

## Data Availability

datasets related to this article will be avail-able upon request to the corresponding author.
